# Transcriptional response of pancreatic beta cells to metabolic stimulation: large scale identification of immediate-early and secondary response genes

**DOI:** 10.1186/1471-2199-8-54

**Published:** 2007-06-22

**Authors:** Dominique A Glauser, Thierry Brun, Benoit R Gauthier, Werner Schlegel

**Affiliations:** 1 Fondation pour Recherches Médicales, University of Geneva, Av. de la Roseraie 64, 1211 Geneva, Switzerland; 2 Department of Cell Physiology and Metabolism, University Medical Center, Rue Michel-Servet 1, 1211 Geneva, Switzerland

## Abstract

**Background:**

Physiological long term adaptation of pancreatic beta cells is driven by stimuli such as glucose and incretin hormones acting via cAMP (e.g. GLP-1) and involves regulated gene expression. Several rapidly inducible immediate-early genes (IEGs) have been identified in beta cells. Many of these IEGs code for transcription factors and have the potential to control the transcription of downstream *target *genes likely involved in long term cellular adaptation. The identity of these *target *genes has not been determined, and the sequence of events occurring during beta cell adaptation is still unclear.

**Results:**

We have developed a microarray-based strategy for the systematic search of *targets*. In Min6 insulin-secreting cells, we identified 592 *targets *and 1278 IEGs responding to a co-stimulation with glucose and cAMP. Both IEGs and *targets *were involved in a large panel of functions, including those important to beta cell physiology (metabolism, secretion). Nearly 200 IEGs were involved in signaling and transcriptional regulation. To find specific examples of the regulatory link between IEGs and *targets*, *target *promoter sequences were analyzed *in silico*. Statistically significant over-representation of AP-1 response elements notably suggested an important role for this transcription factor, which was experimentally verified. Indeed, cell stimulation altered expression of IEG-encoded components of the AP-1 complex, activating AP-1-dependent transcription. Loss and gain-of-function experiments furthermore allowed to validate a new AP-1 regulated gene (*sulfiredoxin*) among the *targets*. AP-1 and *sulfiredoxin *are sequentially induced also in primary cells from rat islets of Langerhans.

**Conclusion:**

By identifying IEGs and their downstream *targets*, this study brings a comprehensive description of the transcriptional response occurring after beta cell stimulation, as well as new mechanistic insights concerning the AP-1 transcription factor.

## Background

In mammals, blood glucose homeostasis is subjected to a tight endocrine control relying notably on insulin-producing beta cells from the pancreas. Beta cells secrete insulin in response to increased glucose concentrations and to hormones released from gut cells, like glucagon-like peptide-1 (GLP-1). In longer term, these physiological signals also control insulin production, stimuli-responsiveness and beta cell mass [1-4]. Over the years, this regulation helps the organism maintaining glucose homeostasis, accommodating changes in diet and insulin demand. Deficient adaptation may lead to diabetes [5,6].

Long term adaptation of beta cell is associated with changes in gene transcription. Several glucose-regulated genes have been identified which are likely involved in the chronic effects of glucose on beta cell mass and function [3,7,8]. Some of these genes are immediate-early response genes (IEGs) rapidly induced upon acute beta cell stimulation with glucose and other stimuli [7,9,10].

IEGs are defined as genes which transcription is regulated without the need for protein *de novo *synthesis. Induction of IEG transcription is triggered by intracellular signals which activate constitutively expressed transcription factors acting on IEG promoters [11,12]. In beta cells, the mechanisms leading to the induction of IEGs by glucose and GLP-1 were shown to involve generation of cAMP, as well as cell depolarization and subsequent Ca^2+ ^signaling, resulting in activation of calmodulin-dependent kinase IV, protein kinase A and extracellular signal-regulated kinase 1/2. These kinases lead to phosphorylation and activation of several transcription factors, like cAMP-responsive element binding protein, serum response factor, and Elk-1 [13-16]. These mechanisms occur rapidly and result, within minutes, in transcriptional regulation of IEGs. Such direct and rapid mechanisms do not however permit to understand regulation of genes for which expression levels change later and over much longer time frames (hours, days), and which are likely relevant for the long term adaptation of beta cell. Other types of mechanisms must be taken into account.

The best studied IEGs in beta cells (e.g. c-*fos*, *egr-1*, *nur77*, c-*myc*) code for transcription factors [7,9,10,17]. It has been proposed that these transcription factors will in turn regulate the transcription of downstream target genes (which will be referred as *targets *throughout this manuscript). The distinction can thus be made between two modes of transcriptional regulation: (i) direct regulation, which is independent of protein synthesis (and concerns IEG induction), and (ii) indirect regulation, which requires preliminary IEG induction (and concerns *target *gene control). A cascade of gene induction, involving upstream IEGs and downstream *targets*, represents an attractive model to explain how beta cell can bridge the time gap between relatively short-lived stimuli and long term cellular adaptation of gene expression. [18]

The aims of the study were, first, to identify *targets *at a large scale in order to evaluate the importance of indirect regulation of gene transcription, second, to define the cellular functions concerned, and third, to verify the regulatory link between specific IEG transcription factors and *targets*.

We have designed a genome-scaled approach to identify glucose and cAMP-induced IEGs and their *targets*. Database annotation searches were applied to infer cellular functions of these genes. *In silico *analysis of *target *gene promoters as well as gain and loss of function experiments were performed to establish the link between specific IEGs and *target *gene induction.

The data reported here suggest that a large part of gene expression induced upon beta cell stimulation is regulated by indirect mechanisms requiring preliminary IEG induction. Functions attributed to gene products cover a large spectrum of cellular processes for both IEG and *targets*, including metabolism, secretion, control of cell growth, signal transduction and regulation of transcription.

## Results

### Identification and validation of glucose- and cAMP-regulated IEGs and *targets*

**Figure 1:**
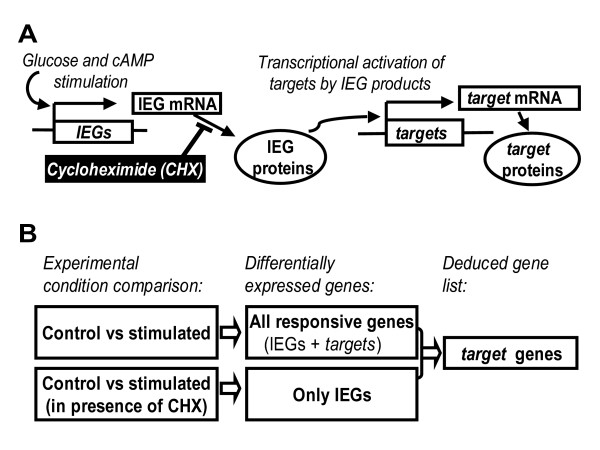
**Strategy to identify glucose and cAMP responsive IEGs and their downstream *targets***. A) IEGs (immediate-early genes) are genes which are transcriptionaly inducible in a protein synthesis independent manner. IEG products regulate in turn the transcription of downstream *targets*. Cycloheximide (CHX), a protein synthesis inhibitor, blocks IEG product synthesis and subsequent activation of *target *transcription. B) Genes induced by glucose and cAMP comprise both IEGs and *targets*. Genes induced by glucose and cAMP in presence of CHX represent only IEGs. Note that CHX is also present in the control condition for this comparison. *Target *genes were deduced by subtraction between the gene lists.

To identify genes regulated by metabolic signals in beta cells, we stimulated Min6 insulin-secreting cells with a combination of elevated glucose (10 mM) and chlorophenylthio-cAMP (cpt-cAMP, 0.2 mM), a membrane-permeant cAMP analogue. Indeed, glucose induction of gene expression is potentiated by gut hormones (such as glucagon-like peptide-1, GLP-1) which raise cAMP [9]. Our initial transcript profile comparison using high density oligonucleotide microarrays identified genes regulated by a 4 hour treatment with glucose and cpt-cAMP (666 up-regulated, 1204 down-regulated; fold change threshold of 1.5). Comparison with profiles obtained in presence of the protein synthesis inhibitor cycloheximide (CHX) allowed classifying each stimuli-responsive gene either as IEG or as *target*. Indeed, by blocking protein neosynthesis, CHX blocks synthesis of IEG products and their downstream regulatory action on *target *transcription. Thus, among the stimuli-responsive genes, those still regulated in presence of CHX were defined as IEGs, while those no longer regulated were defined as *targets *(Figure 1, and Additional file 1).

Among the 1870 regulated genes, 592 *target *genes were identified (Table 1). Thus, a substantial part of the impact produced by metabolic signals on beta cell transcriptional program rely on the indirect action of IEG products.

**Table 1:** Number of IEGs and *target *genes responding to glucose and cAMP identified in the transcript profiling experiment

	**IEGs**	***targets***	**total**
Up-regulated	465	201	666
down-regulated	813	391	1204

**Figure 2:**
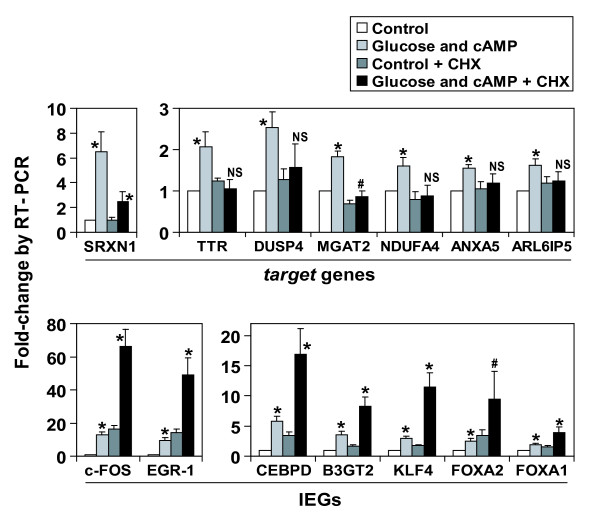
**Validation of IEGs and *targets *by quantitative RT-PCR**. Induction of *targets *is inhibited in presence of CHX, while induction of IEGs is not. Min6 cells cultured at low glucose for 20 hours were stimulated for 4 hours with 10 mM glucose and 0.2 mM cpt-cAMP, in presence or absence of CHX (5 μg/ml, added 45' prior to stimulation). mRNA levels for indicated genes were assessed by quantitative real-time RT-PCR and normalized with 18S rRNA. Results are expressed as mean of fold change compared to control condition (s.d. as error bars, n = 5). *, p < 0.01; #, p < 0.05; NS: non significant vs respective control condition (i.e with or without CHX), by Student T-test.

To validate microarray data, we performed quantitative RT-PCR analysis for a selected set of IEGs and *target *genes. Comparison between fold-change values obtained from microarray or RT-PCR experiments revealed that the microarray data were of excellent quality (mean difference between microarray and RT-PCR lower than 20%; see Additional file 2). Furthermore, the CHX effect was confirmed by RT-PCR. Indeed, *target *regulation by glucose and cAMP was abrogated in presence of CHX (Figure 2). Furthermore, despite a small effect of CHX by itself on all glucose-inducible IEGs examined, these were as strongly stimulated by glucose and cAMP in the presence of CHX as in its absence (Figure 2). Thus RT-PCR data validate the selection of *targets *and IEGs based on the microarray data.

### Functional classification of IEGs and *targets*

**Figure 3:**
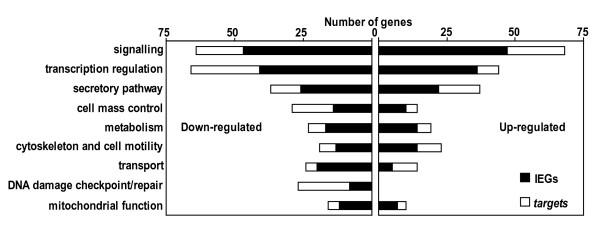
**Functional clustering of glucose and cAMP responsive IEGs and *targets***. From the list of glucose and cAMP regulated transcripts, we retrieved 755 genes with annotations in Swiss-Prot database and clustered them according to functional categories. The diagram depicts the predominant clusters (gathering 534 genes); the 221 remaining genes were found in smaller clusters and are not represented.

To address the functional role of IEGs and *targets *regulated by glucose and cAMP in beta cells, we classified them into functional categories based on the annotations in the Swiss-Prot database (Figure 3) [19]. Of 755 glucose regulated genes with annotations, more than 200 are involved in signaling and transcription regulation. The large number of IEGs involved in these two processes suggests the importance of indirect mechanisms in gene regulation. Moreover, an important fraction of *target *genes are themselves involved in signal transduction and transcriptional regulation.

Other important functional clusters include genes involved in the secretory pathway, the metabolism or the control of cell mass; processes that are known to be regulated by glucose in beta cells [8]. Thus, the response of our Min6 model is in agreement with previous knowledge on the effect of metabolic stimuli on gene expression in beta cells. The genes involved in the secretory pathway concerned with protein synthesis, translocation and folding in the ER, glycosylation, vesicule transport, and exocytosis, are detailed in the Additional file 3. Interestingly, an important cluster of down-regulated genes is involved in DNA damage checkpoint/repair pathways.

Noteworthy, the roles of IEG products are not restricted to transcriptional regulation and cover a large panel of functions (Figure 3 and Additional file 3). Thus, while on the one hand, some IEGs can act indirectly by regulating expression of *target *genes, on the other hand, different IEGs can contribute directly to the functional adaptation of beta cell to metabolic stimulation.

### *Target *promoter analysis reveals the importance of AP-1 transcription factor

**Figure 4:**
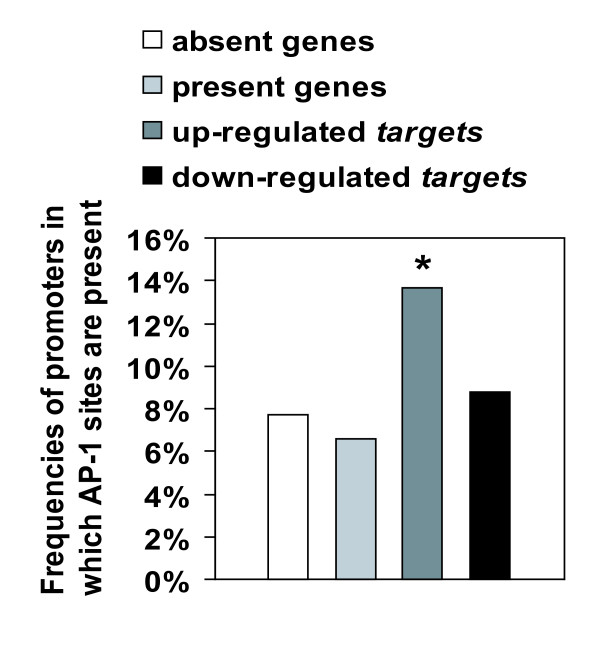
**AP-1 binding sites are over-represented in promoters of up-regulated *targets***. Frequencies of promoters containing at least one AP-1 binding site were determined using TFExplorer predicted regulatory element database. Among the genes displayed on the microarray, *absent genes *were those with undetectable expression in Min6 and *present genes *were those with detectable expression in Min6. *, p < 0.01 by Fisher exact test vs *present genes*.

To establish the link between IEGs that encode transcription factors and their *targets*, we analyzed the promoters of *target *genes. Predicted regulatory elements in *target *gene promoters as well as in control sets of promoter (random sets of promoters from genes expressed or not expressed in Min6 cells) were gathered using the TFExplorer database [20]. We then evaluated over- and under-represented regulatory elements in *targets *promoters compared to control sets. 32 regulatory elements binding specific transcription factors were significantly over-represented in glucose-responsive *target *gene promoters (p < 0.05; Additional file 4). These include notably binding sites for E2F and DP-1 transcription factors found both in up-regulated and down-regulated *targets*. But the most markedly over-represented regulatory element was an AP-1 binding site which was present in 14% of the up-regulated *targets *(Figure 4). Interestingly, this binding sequence was not enriched in down-regulated *targets*. This suggests an important role for AP-1 in transcriptional activation of *target*, and prompted us to investigate its regulation in more details.

### Glucose and cAMP regulate AP-1 complex composition and function

**Figure 5:**
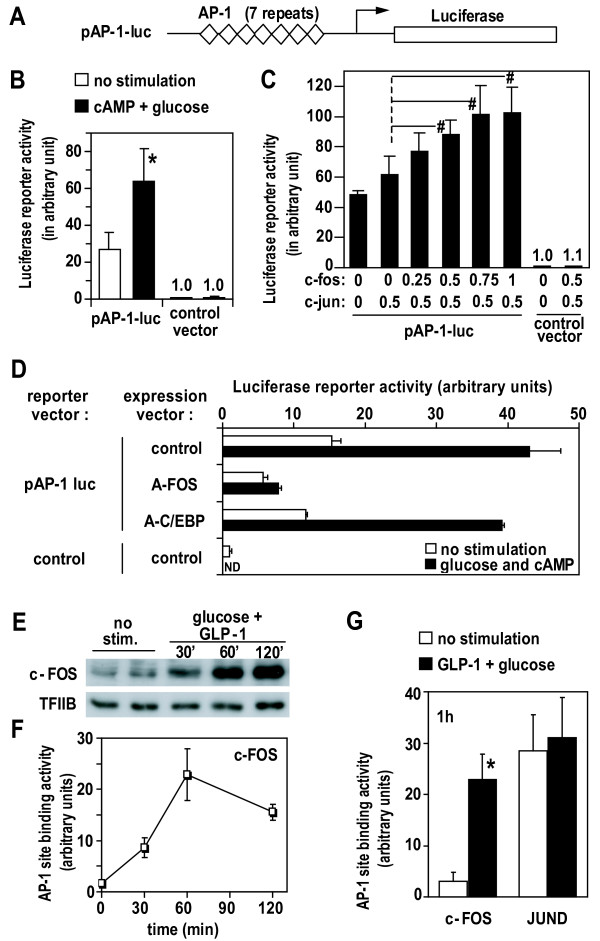
**Glucose and cAMP regulate transcriptional activation by AP-1 through induction of AP-1 component expression**. A) Schematic representation of pAP-1-luc reporter. B) Min6 cells were transfected with pAP-1-luc (or control vector) and maintained at low glucose before stimulation with glucose (10 mM) and cpt-cAMP (0.2 mM) for 6 hours. C) Cells were transfected with AP-1 reporter (or control vector) and indicated quantity (in μg) of expression vector for c-fos and c-jun. D) pAP-1 reporter vector was co-transfected with either A-FOS (a c-FOS dominant negative form), empty vector (control) or A-C/EBP as additional control (dominant negative form of C/EBP, a transcription factor structurally related to c-FOS). Stimulations were performed as under B. E,F,G) Min6 cells cultured at low glucose were stimulated with high glucose (10 mM) and GLP-1 (10 nM) for indicated period of time. Nuclear extracts were analyzed by western (E). Specific binding of c-FOS and JUND to AP-1 sequence was measured in nuclear extracts with an ELISA-like assay (F,G). E, F) representative of two repeated experiments. ^#^, p < 0.05 vs c-Jun alone (n = 3); *, p < 0.01 (n = 4), by Student T-test. Error bars: s.d.; ND: not determined.

AP-1 binds DNA as a dimer composed of proteins of the Fos and Jun families; forming either a Jun/Jun homo-dimer or a Fos/Jun hetero-dimer [21]. Min6 cells maintained at low glucose predominantly expressed c-*jun *and *junD*, indicating that the AP-1 complex is likely composed of Jun/Jun homo-dimer (Table 2). Elevated glucose and cAMP produced a robust 10 fold increase in c-*fos *and *fosB *(IEGs), while c-*jun *and *junD *expression was slightly inhibited (Table 2 and data not shown). These results suggest a potential shift in the composition of the AP-1 complex toward Fos/Jun hetero-dimer and consequently a possible alteration of AP-1 transactivating activity. To test this possibility, we transfected cells with a luciferase reporter gene under the control of an artificial promoter harboring solely AP-1 sites as enhancers (pAP-1-luc; Figure 5A). Cells maintained at low glucose expressed the reporter constitutively, likely reflecting the transcriptional activation by the constitutive (Jun/Jun) AP-1 factors. Despite this relatively high basal level of expression, glucose and cAMP still further stimulated the AP-1 reporter expression significantly (Figure 5B).

**Table 2:** Glucose and cAMP stimulation modulates the expression pattern of genes coding for AP-1 components

**gene**	**mean of microarray signal**		**fold-change**
		
	**control**	**stimulated**	
c-*fos*	107	1'161	10.8*
*fosB*	6	60	10.7*
*fra-1*	5	27	5.3
*fra-2*	71	458	7.0*
c-*jun*	249	205	-1.2
*junB*	59	275	4.6*
*junD*	811	647	-1.2

* significantly and consistently regulated IEGs

Further experiments were performed to determine whether the increased transcriptional activation by AP-1 could be explained by the induction of Fos genes. Min6 cells were co-transfected with pAP-1-luc and expression vectors for c-*fos *and c-*jun*. At constant levels of the c-*jun *vector, increasing amounts of transfected c-*fos *vector led to a gradual increase in reporter gene expression (Figure 5C). Consistently, Fos/Jun hetero-dimers transactivate more efficiently than Jun/Jun homo-dimers (see Additional file 5). Thus, the accumulation of c-*fos *gene product is sufficient to enhance transcription of AP-1 regulated genes.

In addition, we used A-FOS (a dominant negative form of AP-1) to block endogenous AP-1 transcription factor [22]. Results demonstrate that endogenous AP-1 mediates the transcriptional activation of the AP-1 reporter during stimulation with glucose and cAMP (Figure 5D). As control, we used A-C/EBP (a dominant negative form of C/EBP transcription factor, structurally but not functionally similar to AP-1) which did not alter AP-1 reporter transcription neither in basal nor in stimulated conditions.

We then determined if the transcriptional induction of endogenous c-*fos *by glucose and cAMP would effectively change the composition of DNA binding AP-1 complex. Min6 cells were stimulated with glucose and glucagon-like peptide-1 (GLP-1), a physiologically active glucoincretin hormone which raises cAMP in beta cells [1]. Upon stimulation, c-*fos *mRNA induction was followed by a marked increase in nuclear c-FOS protein, as seen by western blot analysis of nuclear extracts (Figure 5E), as well as by immunocytochemistry (not shown). As a result, glucose-induced FOS protein formed functional AP-1 complexes (Figure 5F and 5G). This was shown by ELISA quantification of the components of AP-1 complexes binding to solid phase linked double stranded DNA with the specific AP-1 consensus sequence. The strong correlation between accumulation of FOS in nuclear extracts and its DNA binding activity indicates that newly synthesized FOS protein is efficiently recruited to DNA-binding AP-1 complexes (Figure 5E versus 5F). In contrast to c-FOS, and accordingly to a constant level of expression, JUND in functional AP-1 complexes remained unchanged (Figure 5G).

The data in Figure 5 show that after glucose and cAMP induction of *fos*- and *jun*-like IEGs, the newly synthesized components are recruited to DNA binding AP-1 complexes, notably shifting their composition to Fos/Jun hetero-dimers which are more potent trans-activators than the constitutive Jun/Jun homo-dimers. Qualitative and quantitative changes in the AP-1 complex may explain the increased expression of a large number (14%) of glucose up-regulated *target *genes. This mechanism involves transcriptional induction of IEG and illustrates how IEGs and their downstream *targets *are linked.

### AP-1 regulates the transcription of the *target *gene *sulfiredoxin *(*srxn1*/*npn3*)

**Figure 6:**
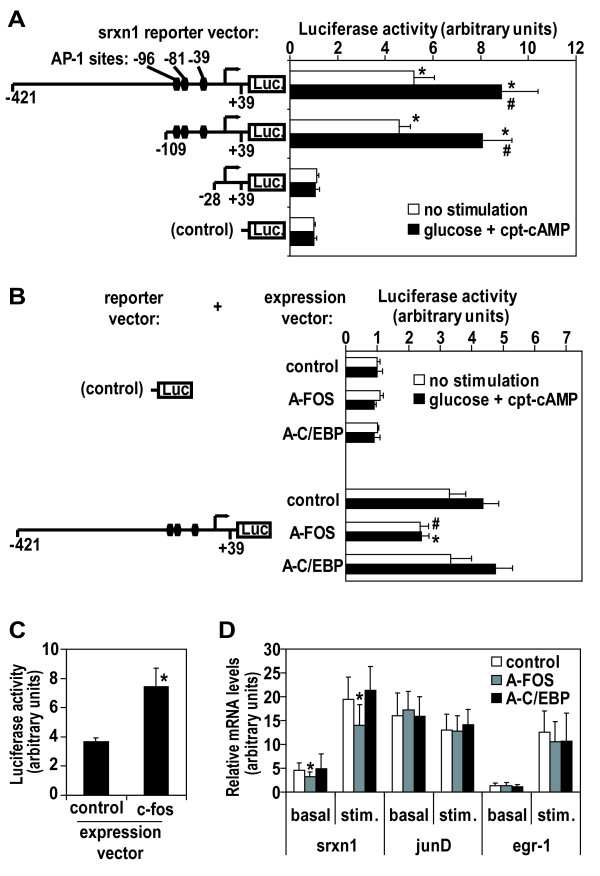
***Srxn1 *is a transcriptional target of AP-1**. A) Depicted srxn1 reporter constructs were transfected in Min6 cells. Results as means of four independent experiments, with s.d. as error bars. *, p < 0.01 vs control; #, p < 0.01 vs corresponding non-stimulated condition, by Student T-test. B) Depicted srxn1 reporter constructs were co-transfected respectively with A-FOS, A-C/EBP, or a control expression vector. Results as means of four independent experiments, with s.d. as error bars. *, #, p < 0.01, respectively p < 0.05 vs control expression vector, by Student T-test. For A) and B), stimulations were performed with 0.2 mM cpt-cAMP and 10 mM glucose for 6 hours. C) srxn1-421/+39pGL3 reporter was cotransfected with c-fos expression vector (as in Figure 5). Results as means of four independent experiments, with s.d. as error bars. *, p < 0.01 vs control expression vector, by Student T-test. D) mRNA levels for *srxn1*, *junD *and *egr-1 *were quantified by RT-PCR in Min6 clones stably transfected with A-FOS, control expression vector and A-C/EBP respectively. Stimulations were performed with 0.2 mM cpt-cAMP and 10 mM glucose for four hours. Four different cell preparations were analyzed for each of at least three clones in each category. Results were pooled and expressed as mean of relative mRNA levels (arbitrary units) with s.d. as error bars. *, p < 0.001 vs control, by Student T-test.

Having demonstrated that glucose and cAMP can activate AP-1 transcription factor, we pursued our investigations to identify which specific *targets *are effectively regulated by AP-1. A candidate approach was undertaken. The most strongly induced *target *gene in our system was *sulfiredoxin *(*srxn1*, also known as *npn3*) and we identified three predicted AP-1 sites in its promoter; located 96, respectively 81 and 39 base pairs (bp) upstream of the transcriptional start site. *Srxn1 *promoter regions from different sizes were then cloned in front of a luciferase reporter gene and these constructs were transfected in Min6 cells. Results of this approach show that a narrow region of 81 bp containing the three AP-1 sites is both sufficient and necessary to *srxn1 *gene transcription in basal and stimulated conditions (Figure 6A). Co-transfection with the A-FOS dominant negative form of AP-1 impaired basal level of *srxn1 *reporter transcription and abolished stimuli responsiveness, indicating that AP-1 was necessary for these effects (Figure 6B). In addition, ectopic expression of c-Fos was sufficient to stimulate *srxn1 *reporter transcription (Figure 6C).

Finally, we established stably transfected Min6 cell lines expressing A-FOS as well as control constructs. The down regulation of AP-1 function in these stable clones was confirmed by assessing the transcriptional activation of a transiently transfected pAP-1-luc reporter (Additional file 6). The expression levels of endogenous *srxn1 *was then evaluated by quantitative RT-PCR (Figure 6D). While *junD *mRNA levels (control) were constant among the different clones, both basal and stimulated *srxn1 *mRNA levels were reduced in A-FOS clones. These effects were not due to a defective stimuli-responsiveness in A-FOS clones since *egr-1 *IEG responded normally. In conclusion, data in Figure 6 show that *srxn1 *is a downstream transcriptional target of AP-1.

### AP-1 and its *target srnx1 *are regulated by metabolic stimuli in primary pancreatic islets

Because some aspects of signaling in cell lines (like Min6) may be altered by the transformation or the long term maintenance in culture, we evaluated if our results could be consistently reproduced in primary cells from isolated islets.

**Figure 7:**
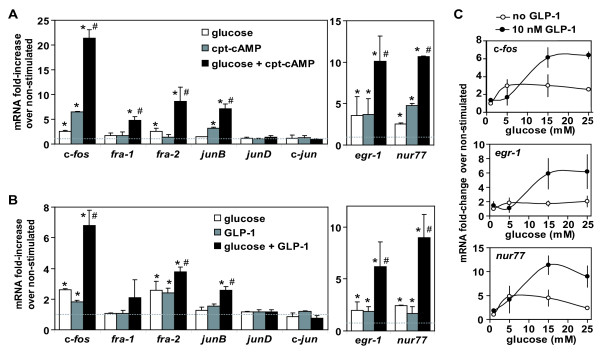
**Induction of IEGs by metabolic stimuli in isolated rat islets**. Rat islets were isolated, cultured and serum deprived at reduced glucose concentration (1 mM) for 20 hours. Stimulation was done for one hour with 0.2 mM cpt-cAMP and/or 25 mM glucose (A); or with 10 nM GLP-1 and/or 25 mM glucose (B). mRNA levels for mentioned genes were quantified in triplicate by real-time RT-PCR, normalized with reference to 18S rRNA, and are shown as fold-increase over non-stimulated controls. Shown are the means of values obtained for three (A) or two (B) independent experiments (error bars = s.d.). Student T-tests were used for statistical analysis; *p < 0.05 vs non-stimulated; # p < 0.05 vs single stimulus conditions. C) Effect of various glucose concentrations on the induction level of IEG expression (after one hour stimulation). Results as mean of at least two independent experiments (s.d. as error bars).

Firstly, we evaluated the effects of metabolic stimuli on expression of several IEG mRNA in isolated rat primary islets (Figure 7). Glucose synergized with cpt-cAMP or with GLP-1 to induce c-*fos*, *fra-1*, *fra-2 *and *junB*, while c-*jun *and *junD *remained unaffected by these stimuli (Figure 7A and 7B). The pattern is identical to what was observed in Min6 (Table 2). Furthermore, we observed induction of *egr-1 *and *nur77 *(two IEGs encoding transcription factors which were induced in Min6), indicating that the parallel between Min6 and primary islets is not limited to AP-1. Interestingly, dose-dependency experiments indicated that maximal stimulation of IEGs was reached in presence of GLP-1 and within a physiological (5 to 15 mM) range of glucose concentrations (Figure 7C). Noteworthy, we obtained similar synergistic activation of IEGs with isolated (FACS sorted) beta cells stimulated with glucose and GLP-1 (Additional file 7), respectively glucose and cpt-cAMP (data not shown). Without excluding induction of IEGs in other islet cell types (like glucagon-secreting cells or somatostatin-secreting cells), these data indicate that IEG mRNA accumulates in response to metabolic stimuli in primary pancreatic beta cells.

**Figure 8:**
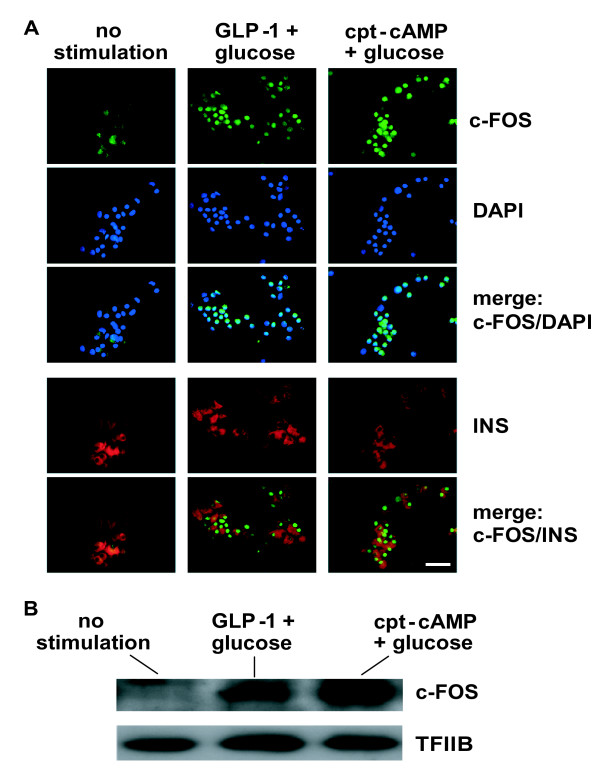
**Accumulation of c-FOS protein in the nuclei of primary beta cells upon metabolic stimulations**. A) Islets were isolated, trypsin digested, cultured and serum deprived at low glucose concentration (1 mM) for 20 hours. After 60 minutes of co-stimulation with 10 nM GLP-1 and 15 mM glucose or 0.2 mM cpt-cAMP and 15 mM glucose, islets (50–100 per condition) were fixed and analyzed by immunofluorescence staining of c-FOS (green) and of insulin (INS, red); nuclei were stained with the DNA reactive DAPI dye (violet). Fluorescence images shown separately for each dye or merged (c-FOS/DAPI; c-FOS/INS) are representative of three different experiments. Bar: 50 μm. B) Islets were isolated, maintained and serum deprived at low glucose concentration (1 mM) for 20 hours, prior to co-stimulation with 10 nM GLP-1 and 15 mM glucose or 0.2 mM cpt-cAMP and 15 mM glucose. After 90 minutes of stimulation, islets (~800 per condition) were trypsin digested, nuclear extracts were prepared and c-FOS expression analyzed by western blotting. TFIIB was used as loading control.

Secondly, we investigated whether changes in IEG mRNA levels were effectively followed by changes in protein levels. c-FOS expression was thus assessed by immunocytochemistry in dispersed islets (Figure 8A) and by western blot analysis in nuclear extracts prepared from intact islets (Figure 8B). Under low glucose conditions, immunofluorescence staining of dispersed islets produced only a faint background signal for c-FOS. Upon co-stimulation by glucose and GLP-1 or cpt-cAMP, expression of c-FOS became strongly apparent. c-FOS protein was mainly detected in nuclei, co-localizing with DAPI staining for DNA. Co-staining for insulin showed that nuclear c-FOS protein was found in beta cells. Western blot analysis confirmed the accumulation of c-FOS protein in the nucleus of primary islet cells. As depicted in Figure 8B, c-FOS protein levels were nearly undetectable in islets maintained at low glucose concentrations. Within 90 minutes of stimulation by GLP-1 and elevated glucose, c-FOS became prominently present in the nuclear extracts. Using cpt-cAMP as co-stimulator with glucose led to a further increase in the c-FOS signal on the western blots. Quantitatively, these data on nuclear c-FOS protein corroborate observations made at the mRNA level, with a strong effect of cpt-cAMP and a more moderate effect of GLP-1 (Figure 8B), and are similar to observations made in Min6 cells (Figure 5E, Additional file 8 and data not shown).

**Figure 9:**
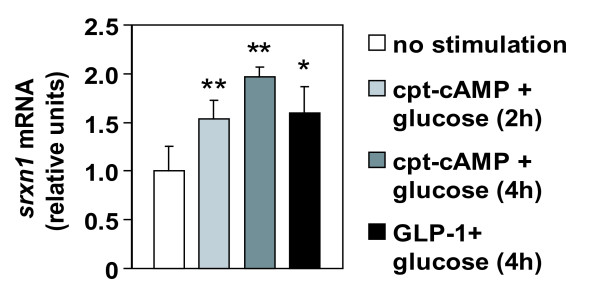
**Induction of *srxn1 *expression by metabolic stimuli in isolated rat islets**. Rat islets were isolated, cultured, and serum deprived at reduced glucose concentration (1 mM) for 20 hours. Stimulation was done for indicated period of time with high glucose (25 mM) plus cpt-cAMP (0.2 mM) or GLP-1 (10 nM). Transcript levels of the AP-1 target gene *srxn1 *were quantified by real-time RT-PCR, normalized with reference to 18S rRNA, and shown as relative values. Shown are the means of values obtained for at least three experiments. Student T-tests were used for statistical analysis; *p < 0.05 vs non-stimulated; ** p < 0.01 vs non-stimulated.

Thirdly, we evaluated whether AP-1 regulation by metabolic stimuli could be functional in islets. To that purpose, expression levels of the newly identified AP-1 target gene *srxn1 *were quantified by RT-PCR in isolated rat islets. As in Min6 cells, co-stimulation with glucose and cpt-cAMP, or glucose and GLP-1, induced significant accumulation of *srxn1 *mRNA (Figure 9).

Altogether, data in Figures 7, 8, and 9 show that a functional AP-1 regulation occurs in primary beta cells in response to metabolic stimuli. This shows the potential physiological relevance of the observations made in Min6, and emphasizes the interest of the dataset obtained with our screening approach in this cell line model.

## Discussion

The present study provides a comprehensive view on the changes in gene transcription that occur within the first hours after pancreatic beta cell stimulation with glucose plus cAMP or incretin hormones. Induced IEGs and their downstream *targets *were identified on a genomic scale. Both IEGs and *targets *code for proteins involved in a large spectrum of cellular functions, including those tightly related to beta cell physiology, suggesting that these inductions may mediate long term cellular adaptation. Statistics on the response elements in *target *promoters pointed towards a predominant role of AP-1 composed of IEG products of the *fos *and *jun *families. The mediator role of AP-1 for the induction of a newly identified specific target (*srxn1*) was substantiated by expressing loss- and gain-of-function mutants of c-*fos*.

### Indirect mechanisms of gene regulation

The metabolic state of a pancreatic beta cell is translated readily and without delay into insulin secretion which is adjusted within minutes to altered glucose stimulation. Such direct translation of metabolic state into regulated expression is not possible for genes which change their expression over much longer time frames (hours, days). The transcriptional regulation of IEGs coding for transcription factors according to beta cell metabolic activity provides a mechanism by which *target *gene transcription can be controlled over longer time frames. Increasing or decreasing the presence of such transcription factors on the promoters of *target *gene will affect the rate of transcription. Here we illustrate this mechanism with the AP-1 transcription factor, the importance of which is indicated by the over-representation of AP-1 binding sites in up-regulated *target *promoters. By modulating the pattern of expression of the different AP-1 complex components (notably through the strong induction of c-*fos*), glucose and cAMP shift the composition of AP-1 dimer. The transcriptional induction of c-*fos *by glucose and cAMP relies on the activation of CaMkinase II and PKA which converge to regulate cis-elements in its promoter (Susini et al., 2000); in addition insulin signaling may also be involved, as reported in other cell systems (Griffiths et al., 1998). The shift in AP-1 composition increases transcriptional activation of an AP-1 reporter gene, as well as at least one endogenous AP-1 target (*srxn1*), showing that it has the potential to affect numerous genes with an AP-1 binding sequence in their promoters. Furthermore, the shift in AP-1 composition (leading to a change in its transactivation potential), as well as the up-regulation of *srnx1 *are observed in cultured intact islets. Together with the observation of IEGs induction in vivo [10], this illustrates the physiological relevance of our observations in the Min6 cell line.

The AP-1 example illustrate the complexity of indirect regulation, since this transcription factor exists in many combinations of JUN and FOS like proteins, some of which are constitutive and some of which can be post-translationally modified. This gives rise to a whole repertoire of AP-1 complexes with differential selectivity for distinct AP-1 binding sites, varying trans-activation potential, and differential interactions with other transcription factors acting on the same promoter [23-25].

It should be mentioned that transcriptional activation, as proposed here with the example of AP-1, is not the only possible mechanism able to explain changes in mRNA levels (as those quantified by microarrays and RT-PCR); IEG products regulating *target *mRNA levels may also be proteins involved in the control of mRNA degradation or in the upstream signaling pathways that modulate the activity of transcription factors and regulators of mRNA stability.

### Limitations

IEGs are defined as genes induced without the need for protein *de novo *synthesis. IEGs are thus identified with the use of protein synthesis inhibitors like CHX (cycloheximide).

Results obtained with CHX have to be considered with some caution. In addition to blocking protein synthesis, CHX may also affect RNA and protein stability as well as signaling pathways [26,27]. Design and interpretation of our microarray experiments aimed to reduce the impact of the non-specific effects of CHX to a minimum. We considered in our gene lists only those genes which responded to stimulation when tested in the absence of CHX; thus, CHX data were only used to separate IEGs from *targets *within the list of stimuli-responsive genes. Furthermore, in the experiments where cells were stimulated in the presence of CHX, induction was assessed against the CHX control conditions. Therefore, in our analysis, side effects of CHX could have caused mis-labeling of responding genes as IEGs or *targets *under very particular circumstances. As these appear relatively unlikely, we are confident that the large majority of genes are correctly listed. Indeed, the detailed induction kinetic of several IEGs and *targets *genes selected from these lists show distinct temporal patterns of induction; the induction of all *targets *(7 genes analyzed) was delayed compared to the rapid induction of the IEGs (4 genes analyzed) [18]. Moreover, in the present study, we were able to verify the predictions about AP-1 based on the *in silico target *gene promoter analysis. Hence, in spite of the limitations linked to using CHX, our microarray data represent a valid starting point to further advance our understanding of the transcriptional response of beta cells to metabolic signals.

### The cellular function of glucose and cAMP regulated genes

Glucose and cAMP responsive genes form large clusters functionally involved in secretion, metabolism, and beta cell mass control (proliferation/apoptosis). Regulation according to the metabolic state of such specific pancreatic beta cell functions is in agreement with previous studies [8,28]. However, a completely novel and unexpected finding is that genes involved in DNA damage checkpoint/repair represent a large down-regulated cluster. This is consistent with the down-regulation of key transcription factors governing this pathway (*p53*, *foxo3*) and some downstream apoptosis effectors like *bbc3*, also known as *puma *(Additional file 1) [29-31]. This raises intriguing questions about the possible implication of DNA damage pathway in the control of beta cell survival by glucose and will require further investigations.

*Srxn1 *gene product sulfiredoxin reactivates peroxiredoxins (H_2_O_2 _scavenging enzymes) by catalyzing the reduction of sulfinic acids formed on peroxiredoxins following exposure to excessive levels of H_2_O_2 _[32]. Recently, sulfiredoxin was also shown to be involved in the reversal of protein glutathionylation [33]. Thus, sulfiredoxin is emerging as an important component in the cellular redox signaling/control systems. To our knowledge, our study provides the first report on the mechanisms of *srxn1 *gene transcriptional regulation in mammals. Regulating the amount of sulfiredoxin in function of cell metabolic stimulation (which is known to be associated with oxidative stress and generation of H_2_O_2 _in beta cells [34]) constitutes a feedback response susceptible to attenuate H_2_O_2 _effects and signaling.

## Conclusion

The present study sheds light on the mechanisms by which beta cell adapts its transcriptional program in response to metabolic and hormonal stimuli known to control secretion of insulin in the short term and proliferation, cell survival, and responsiveness in the long term. Our findings on the role of the AP-1 transcription factor as a mediator for the induction of s*rxn1 *(*sulfiredoxin*) illustrate that IEG transcription factors may effectively relay metabolic signals to regulate transcription of *target *genes in beta cells.

**Figure 10:**
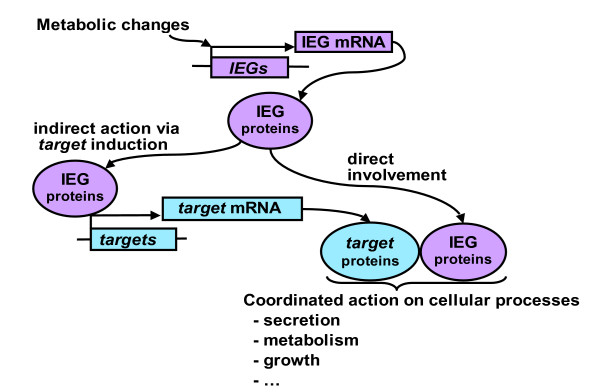
**Cellular adaptation to physiologically relevant stimuli occurring via a combination of direct and indirect modes of transcriptional control**. IEG products have two modes of action in the cellular adaptation to metabolic signals. Some IEG products act indirectly by controlling transcription of *target *genes. Other IEG products are involved directly in regulated cellular processes. A coherent adaptation of these processes requires the combined action of both IEG and *target *gene products.

The large number of induced *target *genes and the wide array of cellular functions concerned suggest that indirect mechanisms of gene regulation (through IEG induction) likely play a substantial role in beta cell adaptation. However, the observation that a significant portion of IEGs codes for proteins that are directly involved in adapted cellular functions shows that direct induction of IEGs transcription complements the indirect mode of gene regulation (Figure 10).

## Methods

### Chemicals

Cycloheximide (CHX), chlorophenylthio-cAMP (cpt-cAMP) and glucagon-like peptide-1 (GLP-1) (7–37, human) were purchased from Sigma (Buchs, Switzerland).

### Min6 cell culture and incubations

Min6 B1 cells [35] (generously provided by Dr. Philippe Halban, Dept of Development and Medical Genetics, Medical faculty of Geneva University) (passage 15–25) were cultured in DMEM supplemented with 15% fetal calf serum, 25 mM glucose, 71 μM 2-mercaptoethanol, 100 units/ml penicillin, 100 μg/ml streptomycin and 50 μg/ml gentamycin. Medium was changed to low glucose medium (same as above with 1% FCS and 1 mM glucose) 20 hours before a 4 hour stimulation with glucose (10 mM) and cpt-cAMP (0.2 mM). When used, CHX (5 μg/ml) was added 45' prior to stimulation.

### Islet and primary beta cell isolation, culture and incubations

7-Week-old male Wistar rats (~250 g) were purchased from Elevage Janvier (Le Genest-St-Isle, France). Pancreatic islets were isolated by collagenase digestion, handpicked, and maintained in 11.1 mM glucose/RPMI 1640 (Invitrogen) supplemented with 10% fetal calf serum (Amimed, BioConcept Allschwil, Switzerland), 100 units/ml penicillin, 100 μg/ml streptomycin and 50 μg/ml gentamycin (Sigma).

For the induction experiments, islets were maintained for 48 hours after isolation and serum deprived for 20 hours in low glucose medium (1 mM glucose RPMI 1640 (Invitrogen), 0.1% BSA and same antibiotics as above). Consecutively, islets were stimulated with glucose, GLP-1 or cpt-cAMP as detailed in the respective figure legends.

For beta cell purification, islets were trypsin digested and FACS sorted as earlier described [36]. Beta cells were maintained in 11.1 mM glucose/Dulbecco's modified Eagle's medium (DMEM) (Invitrogen) supplemented with 10% fetal calf serum and same antibiotics as above, for five days to permit re-aggregation in small clusters. 20 hours before stimulation cells were serum deprived in low glucose medium (1 mM glucose/DMEM supplemented with 0.1% BSA, and same antibiotics as above).

### RNA preparation and microarray analysis

All the stimulations were performed at the same time (in parallel), with an unique batch of cells plated at uniform confluence. For each experimental condition, transcript profiles were established for three different preparations of total RNA made using RNeasy Micro Kit (Qiagen). Labeled cRNA synthesis, hybridization to the arrays and scanning were essentially performed as earlier described [37]. Affymetrix Mouse Genome 430 2.0 oligonucleotide array (containing probe features for 45'101 transcripts) were used. Fluorescence signals from the arrays were analyzed with Affymetrix software GCOS for normalization and calculation of gene-expression values [38,39].

### Criteria to define differentially expressed transcripts

The definition of differentially expressed transcripts between two experimental conditions was based on three criteria: concordance between replicates, statistical significance and fold-change cutoff. The strategy to evaluate the concordance of an effect in two different experimental conditions was the following: each replicate of one condition was compared to each replicate of the other, resulting in 9 pairwise comparisons. Transcripts were considered as differentially expressed if their levels changed in the same direction in at least 7/9 comparisons. The second criterion was a p value by Welch T test below 0.05 when comparing signal values in two experimental conditions. Finally, the third criterion was a minimal fold-change values of 1.5.

### Criteria to delineate IEGs and *targets*

We considered genes that were differentially expressed in *control *(no stimulation) vs *stimulated *(glucose + cAMP) condition; these representing glucose and cAMP regulated genes. We divided this list of genes into two lists: IEGs and *targets*. To do so, different criteria were used.

#### Main criterion

The main criterion was the responsiveness in presence of CHX. From the initial list of regulated genes, we considered genes that were differentially expressed in *control+CHX *(no stimulation in presence of CHX) vs *stimulated+CHX *(glucose + cAMP in presence of CHX) conditions; these represent IEGs (genes that respond to glucose and cAMP in presence of CHX). The rest of the genes (that were initially found to respond to glucose and cAMP but were not regulated in presence of CHX) were defined as *targets*. This single criterion is very stringent for the definition of IEGs, but may lead, on the other hand, to a high level of false positive in the *target *category. This is particularly unsuitable, notably for the validity of *target *promoter sequence analysis. Thus it was necessary to introduce more criteria to increase the quality of *target *list.

#### Criteria to increase *target* list quality (secondary criteria)

Some genes responded to CHX alone. If CHX produces more effect than glucose, it can mask the effect of glucose in presence of CHX (saturating regulation by CHX). For this reason, IEGs can be falsely considered as *targets *with the main criterion (here-above). Thus we excluded from up-regulated *target *gene list, the genes for which expression was higher in either *control+CHX *or *stimulated+CHX *conditions compared to the *stimulated *condition. Similarly, we excluded from down-regulated *target *gene list, the genes for which expression was lower in either *control+CHX *or *stimulated+CHX *conditions compared to the *stimulated *condition. Finally, we excluded from the *target *list, genes for which the mean signal difference between *stimulated+CHX *and *control *represented more than 25% of the signal in the *stimulated *condition. Genes excluded from the *target *list by these secondary criteria were attributed to the IEG list.

### Quantitative real-time RT-PCR

Each total RNA sample was reverse-transcribed in triplicate with random hexamers as primers and Omniscript reverse transcriptase (Qiagen). Quantitative real-time PCR were performed with the SYBR Green system as described in Brun *et al*. [40]. Primers were provided by Microsynth (Balgach, Switzerland) and their sequences are presented in Additional file 9. For normalization, 18S RNA was quantified in each sample using 0.3× 18S rRNA Predeveloped Assay Reagent and 1× TaqMan^® ^Universal PCR Master Mix (Applied Biosystems).

PCR amplicons were quality controlled and all displayed a single homogeneous melting curve as well as the correct size on 2% agarose gels. A cDNA serial dilution standard curve was added to the microtiter plate of each amplification reaction to calibrate each relative quantification in function of PCR amplification efficiency.

### Promoter analysis

TFExplorer predicted regulatory element database [20] was used to map regulatory elements in promoters (from -1000 bp to +300 bp from transcription start site) (accessed on June 17th 2005 [41]). We analyzed promoters of *target *genes (132 up-regulated gene promoters, 239 down-regulated gene promoters) and of two control sets of promoters from genes randomly chosen among those present (detectable in Min6 cells, 1188 promoters) or those absent (undetectable in Min6 cells, 1164 promoters). For each promoter set (up-regulated *targets*, down-regulated *targets *and controls) we counted the number of promoters (Hit numbers) in which a given regulatory element was present (at least once). We calculated the frequencies for any given regulatory element within each set, and evaluated the statistical significance of the difference to the control sets by Fisher exact test.

### Nuclear extract preparation and DNA binding assay

Nuclear protein extracts were prepared according to the protocol of Schreiber *et al*.[42]. The detection of c-FOS and JUND specific binding to AP-1 site was made with the ELISA-like *TransFactor Kit Inflammation II *(BD Biosciences AG, Switzerland) according to supplier instructions except that the colorimetric detection step was replaced by a chemiluminescent one. Briefly, after initial blocking, 12 μg of nuclear extracts were incubated 60 minutes in AP-1 or STAT consensus oligo coated 96-well plates. Plates were then washed three times, incubated 60 minutes with primary antibodies (anti-c-FOS or anti-JUND), washed three times and incubated 30 minutes with HRPO-anti-rabbit-IgG secondary antibody (Transduction Laboratories) (1:10'000). After final four washes, 100 μl of 1× ECL HRP substrate (Cell Signaling Technology) were added to each well and light emission measured three times with a FLUOStar OPTIMA (BMG LABTECH GmbH). Binding to coated STAT oligo and competition with soluble AP-1 oligo were used to check binding specificity. Results were expressed in arbitrary units of DNA binding after normalization by values of no template controls (NTC) for each independent experiment.

### *Srxn1 *reporter construction

*Srxn1 *promoter regions of three different sizes (-421/+39; -109/+39; -28/+39 from the transcriptional start site) were amplified by PCR. Primer were designed from sequences found in ENSEMBL database (entry: ENSMUST00000041500) with addition of 5' flanking residues to create restriction sites (XhoI for forward primers, HindIII for the reverse primer; allowing directional insertion). Three different forward primers were used srxn1-421, AA**CTCGAG**AGACAGCGCTGGGATCCAA; srxn1-109, AA**CTCGAG**GGCCTGAGTCACCACGCT; srxn1-28, AA**CTCGAG**CGTCCATTGAGCGCATCG (XhoI site in bold). A single reverse primer was used srxn1+39: GATT**AAGCTT**CTGACCTAGCTGCCCACTGCC (HindIII site in bold). PCR products were initially cloned into pGEMT-easy vector (Promega) using Takara mighty mix DNA ligation kit (Takara Bio Inc.) and sequentially restriction digested with HindIII and XhoI (Roche). Inserts of respective expected sizes were cloned into pGL3enhancer vector (Promega) that had been previously restriction digested with the same enzymes and treated with alkaline phosphatase (Roche). Construction sequences were verified by the Dye Terminator sequencing technique using Rvprimer3 (CTAGCAAAATAGGCTGTCCC) at the DNA sequencing facility of Geneva University Medical Center.

### Luciferase reporter analysis

0.5 μg PathDetect^® ^cis-Reporting System pAP-1-Luc or pCIS CK (negative control) plasmids (Stratagene Europe, Amsterdam Zuidoost, The Netherlands) were co-transfected with 0.5 μg of Renilla luciferase plasmid (for normalization) (Promega, Luzern, Switzerland) using Lipofectamine 2000 reagent (Invitrogen) according to supplier's instructions. In the ectopic expression experiment, pMSCV-c-Fos (c-Fos expression vector) and/or pMSCV-c-JunFlag (c-Jun expression vector) [43] (both generously provided by Dr. Gerald Thiel, University of Saarland Medical Center, Germany) were cotransfected at various concentrations (see figure legends). Luciferase activity measurement was performed 24 hours after the transfection as previously described [14]. In stimulation experiments, cells were transfected with reporter vectors, maintained for 20 hours in culture medium, changed to low glucose medium for additional 20 hours, and stimulated for 6 hours with 10 mM glucose and 0.2 mM cpt-cAMP (in triplicate).

### AP-1 loss-of-function experiments

pCMV500 (control), pCMV500-A-FOS and pCMW500-A-C/EBP (kind gift of Dr. Charles Vinson, National Cancer Institute, Laboratory of Metabolism, Bethesda, MD, USA) were used in transient co-transfection with reporter constructs or for establishment of stable transfectant Min6 clones. In the latter case, after transfection, 400 mg/l G418 were added to culture medium for a selection period of one month. Clones were picked-up and grown in culture medium supplemented with 200 mg/l G418. A decrease in AP-1 reporter was specifically found in A-FOS clones transiently transfected with pAP-1-luc (Additional file 6). At least three different clones for each construct were used in the experiments.

### Western blotting and immunocytochemistry

Nuclear extracts (15 μg) were resolved on SDS-PAGE (10% gel) and subject to immunoblot analysis as earlier described [44]. Primary antibodies were rabbit anti-c-FOS (1:1'000, sc-52) and anti-TFIIB (1:10'000, sc-225) (Santa Cruz Biotechnology, Inc.). For immunofluorescence studies, partially trypsin dispersed rat islets were cultivated and pre-incubated in low glucose RPMI 1640 medium as described above for intact islets. After stimulation, cells were subjected to cytospin on SuperFrost^®^Plus slides (Menzel GmbH and Co KG, Braunschweig, Germany) and fixed in 4% paraformaldehyde pH 7.0 for 30 minutes at room temperature. After three PBS washes and two incubations with boiling 10 mM citrate pH 6.0 for two minutes, cells were permeabilized with 0.2% Triton PBS for 15 minutes. Primary antibody for c-FOS (rabbit anti-c-FOS, 1:200, sc-52, Santa-Cruz Biotechnology) and mouse anti-insulin (1:1000, I-2018, Sigma) were diluted in 0.05% triton PBS and used for an overnight incubation. After three washes, cells were incubated one hour with secondary antibodies (alexa-488 labeled anti-rabbit-IgG and alexa-568 labeled anti-mouse-IgG (both 1:300; Molecular Probes)). After washings, cells were incubated three minutes in 5 mg/ml 4',6-Diamidino-2-phenylindol (DAPI), washed three times and mounted in Dakocytomation fluorescent mounting medium (DakoCytomation AG, Untermüli, Switzerland). Images were acquired with a Zeiss Axiocam Imaging System (Bioimaging Core Facility, Medical Faculty, Geneva University).

### Numbers of repeated experiments

In the figure legends, n represents the number of repeated experiments. This corresponds to different cell preparations (in the case of islets, preparation from different rats).

## Authors' contributions

DAG conceived the study, designed and performed the experiments, analyzed the results, and drafted the manuscript. TB participated in the experiments with rat islets and helped to draft the manuscript. BRG contributed to experiment design and helped to draft the manuscript. WS conceived the study, designed experiments, and drafted the manuscript. All authors read and approved the final manuscript.

## Accession number

The microarray dataset has been submitted to Array Express database [45] under accession number E-TABM-141.

## Supplementary Material

Additional file 1 **Glucose and cAMP responsive IEGs and *target *genes**. Table presenting the full list of glucose and cAMP responsive IEGs and *target *genes. Click here for file

Additional file 2 **Validation of microarray results by RT-PCR**. Table comparing the fold-change values obtained by microarray versus RT-PCR. Click here for file

Additional file 3 **Glucose and cAMP regulated genes involved in secretion**. Table presenting a list of glucose and cAMP regulated genes involved in secretion. Click here for file

Additional file 4 ***Target *promoter sequence analysis**. Table presenting all the significantly over-. and under-represented regulatory elements. Click here for file

Additional file 5 **Increased transactivation by Fos/Jun compared to Jun/Jun AP-1 dimer**. Figure presenting the results of co-transfection experiments. Click here for file

Additional file 6 **Down-regulation of AP-1 activity in A-FOS stable clones**. Figure presenting the results of transfection experiments. Click here for file

Additional file 7 **Induction of IEGs in purified primary beta cells**. Figure presenting the results of IEG transcript quantification in FACS-sorted primary beta cells. Click here for file

Additional file 8 **Accumulation of c-FOS protein in the nucleus of Min6 cells following stimulation**. Figure presenting the results of Western blot and immunocytochemistry analyses. Click here for file

Additional file 9 **Primer sequences**. Table presenting the sequences of the primers used in the study. Click here for file
