# Supplementary material for: Transcriptional response of pancreatic beta cells to metabolic stimulation: large scale identification of immediate-early and secondary response genes
Source: BMC Mol Biol. 2007 Jun 22;8:54. doi: 10.1186/1471-2199-8-54 (PMC1914353; doi:10.1186/1471-2199-8-54)
Supplement: Additional file 2 — Validation of microarray results by RT-PCR. Table comparing the fold-change values obtained by microarray versus RT-PCR. [file 1471-2199-8-54-S2.pdf]

Additional file 2: Validation of microarray results by quantitative RT-PCR

Category	Gene symbol	Description	Fold-change*	
			microarray	RT-PCR
IEGs	*EGR-1*	early growth response 1	13.30	9.59
	c-FOS	FBJ osteosarcoma oncogene	10.80	13.01
	FOSB	FBJ osteosarcoma oncogene B	10.72	9.01
	CEBPD	CCAAT/enhancer binding protein (C/EBP), delta	8.84	5.82
	B3GALT2	UDP-Gal:betaGlcNAc beta 1,3-galactosyltransferase, polypeptide 2	4.67	3.56
	KLF4	Kruppel-like factor 4 (gut)	3.67	2.95
	IRS1	insulin receptor substrate 1	3.40	3.56
	MAN2A1	mannosidase 2, alpha 1	2.41	2.18
	FOXA1	forkhead box A1	2.14	1.88
	FOXA2	forkhead box A2	2.03	2.47
targets	*SRXN1*	sulfiredoxin (neoplastic progression 3)	5.49	7.99
	DUSP4	dual specificity phosphatase 4	2.56	2.19
	SIAT8E	ST8 alpha-N-acetyl-neuraminide alpha-2,8-sialyltransferase 5	2.00	2.34
	MGAT2	mannoside acetylglucosaminyltransferase 2	1.94	1.83
	ARL6IP5	ADP-ribosylation factor-like 6 interacting protein 5	1.70	1.61
	TTR	transthyretin	1.69	2.07
	ANXA5	annexin A5	1.69	1.55
	SLC35A2	solute carrier family 35 (UDP-galactose transporter), member 2	1.60	1.53
	DAD1	defender against cell death 1	1.58	1.50
	NDUFA4	NADH dehydrogenase (ubiquinone) 1 alpha subcomplex, 4	1.55	1.60
	TRP53	transformation related protein 53 (p53)	-1.85	-1.91
	NGLY1	N-glycanase 1	-1.91	-2.08
	BBC3	Bcl-2 binding component 3 (PUMA)	-2.28	-2.75
non-regulated gene	*JUND1*	Jun proto-oncogene related gene d1	-1.19	-1.30

*For genes with more than one probe set on the microarray, the mean fold-change value is shown. RT-PCR results as mean of fold-change after normalization with 18S rRNA content in each sample (n =5).
