# Supplementary material for: Transcriptional response of pancreatic beta cells to metabolic stimulation: large scale identification of immediate-early and secondary response genes
Source: BMC Mol Biol. 2007 Jun 22;8:54. doi: 10.1186/1471-2199-8-54 (PMC1914353; doi:10.1186/1471-2199-8-54)
Supplement: Additional file 4 — Target promoter sequence analysis. Table presenting all the significantly over-. and under-represented regulatory elements. [file 1471-2199-8-54-S4.pdf]

Additional file 4: *Target* promoter sequence analysis: all over-. and under-represented regulatory elements

Target regulation	Representation (vs control)	Matrix in TRANSFAC	Transcription Factors	Hit numbers		Frequency		Odds ratio	Fisher exact test
				Target set	Control set	*Target* set	Control set		
**Up-regulated *target* promoters**	**Over-represented regulatory elements**	**V$AP1_Q4**	**AP-1**	**18**	**78**	**14%**	**7%**	**2.08**	**0.0048**
		V$E2F_02	E2F, E2F-1, E2F-2, E2F-3, E2F-4, E2F-5	11	37	8%	3%	2.68	0.0058
		V$E2F1DP1RB_01	Rb:E2F-1:DP-1	11	37	8%	3%	2.68	0.0058
		V$E2F_Q3	E2F	5	10	4%	1%	4.50	0.012
		V$E2F4DP1_01	E2F-4:DP-1	5	10	4%	1%	4.50	0.012
		V$E2F_03	E2F, E2F-1	8	29	6%	2%	2.48	0.02557
		V$MYOGNF1_01	NF-1, NF-1A1, NF-1B1, NF-1B2, NF-1C2, NF-1/L, NF-1/Red1, NF-1X	12	54	9%	5%	2.00	0.026
		V$E2F_Q4	E2F	15	75	11%	6%	1.80	0.0283
		V$HTF_01	HTF	2	1	2%	0%	18.00	0.031
		V$E2F_Q3_01	E2F, E2F+E4, E2F-1, E2F-3, E2F-4, DP-1	9	38	7%	3%	2.13	0.038
		V$E2F1_Q4_01	E2F, E2F+E4, E2F-1, E2F-3, E2F-4, DP-1	9	38	7%	3%	2.13	0.038
		V$E2A_Q2	E12, E47, ITF-1, MRF4, MyoD, myogenin	9	40	7%	3%	2.03	0.049
	**Under-represented regulatory elements**	V$MEF2_Q6_01	MEF-2, aMEF-2, MEF-2C, MEF-2DAB	2	68	2%	6%	0.26	0.021
		V$COREBINDING FACTOR_Q6	core-binding	3	72	2%	6%	0.38	0.0458
		V$CREB_Q4_01	CREB, deltaCREB, CREMtau, CREMalpha, CREMbeta, CREMgamma, CREMtaualpha, CREMtau1, CREMtau2, CREBbeta	3	71	2%	6%	0.38	0.049
		V$ELK1_01	Elk-1	2	58	2%	5%	0.31	0.049
**Down-regulated *target* promoters**	**Over-represented regulatory elements**	V$E2F1DP1_01	E2F-1:DP-1	22	55	9%	5%	1.99	0.0053
		V$E2F1_Q6	E2F-1	67	249	28%	21%	1.34	0.011
		V$SREBP1_01	SREBP-1a, SREBP-1b, SREBP-1c, SREBP-1	9	16	4%	1%	2.80	0.015
		V$E2F_Q6	E2F	48	169	20%	14%	1.41	0.015
		V$E2F1DP2_01	E2F-1:DP-2	17	45	7%	4%	1.88	0.021
		V$E2F4DP2_01	E2F-4:DP-2	17	45	7%	4%	1.88	0.021
		V$OCT1_Q6	POU2F1, POU2F1a	52	196	22%	16%	1.32	0.033
		V$PIT1_Q6	POU1F1, POU1F1a, POU1F1b, POU1F1c	18	52	8%	4%	1.72	0.034
		V$E2F1_Q6_01	E2F-1	20	60	8%	5%	1.66	0.034
		V$AR_01	AR	3	2	1%	0%	7.46	0.035
		V$USF_Q6	USF, USF1, USF-1, USF2, USF-1:USF-2	27	89	11%	7%	1.51	0.037
		V$HNF3B_01	HNF-3B	13	34	5%	3%	1.90	0.038
		V$PPARG_03	PPAR-gamma, PPAR-gamma1, PPAR-gamma2	22	70	9%	6%	1.56	0.043
		V$MMEF2_Q6	MEF-2	7	14	3%	1%	2.49	0.0477
	**Under-represented regulatory element**	V$CMYB_01	c-Myb	0	31	0%	3%	0.00	0.004

TFExplorer predicted regulatory element database was used to map regulatory elements in promoters (from -1000 bp to +300 bp from transcription start site). We analyzed promoters of *target* genes (132 up-regulated gene promoters, 239 down-regulated gene promoters in TFExplorer database) and of a control set of promoters from genes randomly chosen among genes that were expressed in Min6 cells (1188 promoters). For each gene promoter set (up-regulated *target*, down-regulated *target* and control) we counted the number of genes (Hit numbers) for which a given regulatory element was present in the promoter. We calculated the frequencies for any given regulatory element, and evaluated the statistical significance of difference to control set by Fisher exact test. The tables present all results with p value < 0.05. Regulatory elements are referred to their matrix name in the TRANSFAC database.
