# Supplementary material for: Transcriptional response of pancreatic beta cells to metabolic stimulation: large scale identification of immediate-early and secondary response genes
Source: BMC Mol Biol. 2007 Jun 22;8:54. doi: 10.1186/1471-2199-8-54 (PMC1914353; doi:10.1186/1471-2199-8-54)
Supplement: Additional file 5 — Increased transactivation by Fos/Jun compared to Jun/Jun AP-1 dimer. Figure presenting the results of co-transfection experiments. [file 1471-2199-8-54-S5.pdf]

Additional file 5

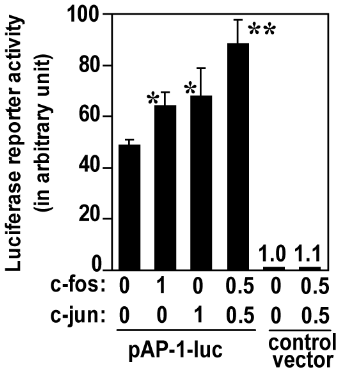

Increased transactivation by Fos/Jun compared to Jun/Jun AP-1 dimer

Min6 cells were transfected with pAP-1-luc reporter (or control vector) and indicated quantity (in µg) of expression vector for c-Fos and c-Jun. *, $p<0.05$ vs no expression vector condition. **, $p<0.05$ vs c-Jun alone, by Student T-test (n=3).
