# Supplementary material for: Transcriptional response of pancreatic beta cells to metabolic stimulation: large scale identification of immediate-early and secondary response genes
Source: BMC Mol Biol. 2007 Jun 22;8:54. doi: 10.1186/1471-2199-8-54 (PMC1914353; doi:10.1186/1471-2199-8-54)
Supplement: Additional file 6 — Down-regulation of AP-1 activity in A-FOS stable clones. Figure presenting the results of transfection experiments. [file 1471-2199-8-54-S6.pdf]

Additional file 6

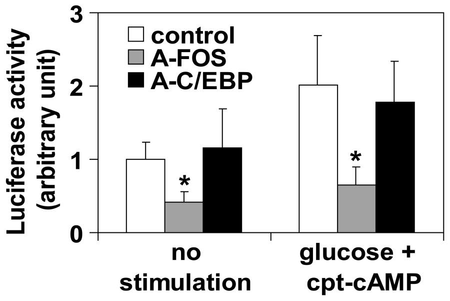

Down-regulation of AP-1 activity in A-FOS stable clones

Clones stably transfected with A-FOS and control constructs were transiently transfected with pAP-1-luc reporter and stimulated with 10 mM glucose plus 0.2 mM cpt-cAMP for 6 h. Three different experiments were performed for each of at least three clones in each category. Results were pooled and expressed as mean of relative luciferase activity (arbitrary units) with s.d. as error bars. *, $p<0.01$ vs control and vs A-C/EBP, by Student T-test.
