# Supplementary material for: Transcriptional response of pancreatic beta cells to metabolic stimulation: large scale identification of immediate-early and secondary response genes
Source: BMC Mol Biol. 2007 Jun 22;8:54. doi: 10.1186/1471-2199-8-54 (PMC1914353; doi:10.1186/1471-2199-8-54)
Supplement: Additional file 7 — Induction of IEGs in purified primary beta cells. Figure presenting the results of IEG transcript quantification in FACS-sorted primary beta cells. [file 1471-2199-8-54-S7.pdf]

Additional file 7

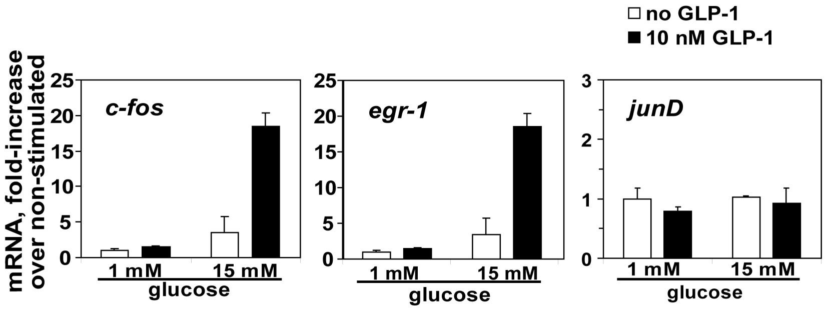

Induction of IEGs in purified primary beta cells

FACS sorted beta cells (from dispersed islets) were cultured for five days to permit their aggregation in small clusters. Cells were serum deprived at low glucose concentration (1 mM) for 24 hours and stimulated with 15 mM glucose and/or 10 nM GLP-1 for 30 minutes. c-*fos, egr-1* and *junD* mRNA levels were quantified in triplicate by real-time RT-PCR, normalized with reference to 18S rRNA and shown as fold-increase over control conditions. Shown as mean of triplicates (error bar = s.d.).
