# Supplementary material for: Transcriptional response of pancreatic beta cells to metabolic stimulation: large scale identification of immediate-early and secondary response genes
Source: BMC Mol Biol. 2007 Jun 22;8:54. doi: 10.1186/1471-2199-8-54 (PMC1914353; doi:10.1186/1471-2199-8-54)
Supplement: Additional file 8 — Accumulation of c-FOS protein in the nucleus of Min6 cells following stimulation. Figure presenting the results of Western blot and immunocytochemistry analyses. [file 1471-2199-8-54-S8.pdf]

Additional file 8

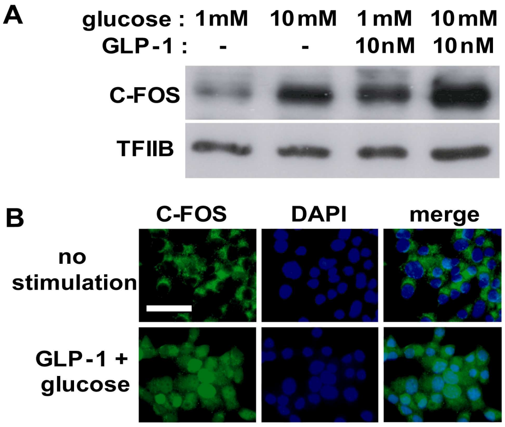

Accumulation of c-FOS protein in the nucleus of Min6 cells following stimulation

MIN6 cells were cultured for 20 hours at low glucose (1 mM) prior to stimulation with 10 nM GLP-1 and/or 10 mM glucose. A) After 90 minutes of stimulation, nuclear extracts were prepared and analyzed by western blotting with a c-FOS specific antibody. The same membrane was then stripped and revealed with anti-TFIIB antibodies as a loading control. B) MIN6 cells were grown on glass coverslips and stimulated for 60 minutes with 10 nM GLP-1 and 10 mM glucose. Immunofluorescence for c-FOS and DAPI staining were performed and shown as described in Figure 8A. Similar results were observed in three independent experiments. Bar: 50 µm. Extranuclear background staining for the green channel is likely due to aspecific binding of c-FOS antibody to an unknown cytosolic component. Indeed, we were unable to detect c-FOS in cytosolic extracts by western analyses, but the antibody detected aspecific bands of different sizes (not shown).
