# Supplementary material for: Transcriptional response of pancreatic beta cells to metabolic stimulation: large scale identification of immediate-early and secondary response genes
Source: BMC Mol Biol. 2007 Jun 22;8:54. doi: 10.1186/1471-2199-8-54 (PMC1914353; doi:10.1186/1471-2199-8-54)
Supplement: Additional file 9 — Primer sequences. Table presenting the sequences of the primers used in the study. [file 1471-2199-8-54-S9.pdf]

Additional file 9: Primer sequences

**Gene**	**Forward primer sequence**	**Reverse primer sequence**
ANXA5	TGGATGCTCAGGCATTGTT	GTACTTGTCAAACACTCTTCTTAAATGAGA
ARL6IP5	GTGGCTGCCATGATGATTT	GGCGGAGGATGTCTTTATTGT
B3GT2	CAGACCTGCCTCCTAGACATAACTA	CATAACCAGTTCCTGAGCAGAA
BBC3	CGGCGGAGACAAGAAGA	CACCTAGTTGGGCTCCATTT
CEBPD	CGCAGACAGTGGTGAGCTT	CGCACAGCGATGTTGTT
c-*FOS*	TGACAGATACGCTCCAAGCG	TGGCAATCTCGGTCTGCA
c-*JUN*	GAAACGACCTTCTACGACGAT	GAATCTTAGGGTTACTGTAGCCGTA
DAD1	GCTGCAGTTCGGCTACTGT	TTGGAAGTCCGCCTTGTT
DUSP4	TGCGCTCTGGCCTCTACT	CCACCTTTAAGCAGGCAGAT
EGR-1	GCACCTGACCACAGAGTCCT	GGGAGAAGCGGCCAGTA
FOSB	CTGTCTTCGGTGGACTCCTT	GGGCCATGGAAGAGATGA
FOXA1	GCAACGACTGGAACAGCTACTA	GGCGTAGGACATGTTGAAGGAA
FOXA2	TCCCTTTCTACCGGCAGAA	TCTCACACTTGAAGCGCTTCT
FRA-1	CAGGCCCTGTGAGCAGAT	CTTTCTTCGGTTTCTGCACTT
FRA-2	AGCAGAAGTTCCGGGTAGATAT	ATCACTGTGGGCTGTACCAT
IRS1	GCTCCAGTGAGGATTTAAGCA	GAAGACGTGAGGTCCTGGTT
JUNB	GGCTTTCTATCACGACGACTCTTA	CTAAGGTGGGTTTCAGGAGTTT
JUND	CGAGCAGCATGCTGAAGA	GCCGACCCTGGTTTCAA
KLF4	GAGGAGCCCAAGCCAAA	GGTTTCTCGCCTGTGTGAGT
MAN2A1	GATGTGCAGATGTTGGATGTTTA	CTTCAACCAACCTGGGTCAT
MGAT2	GACTGTGGTATGCATCACAAGAA	AATGGCTGCCATAGGAAACTT
NDUFA4	TCGGGCAAGCCAAGAA	CCAAGCGCATCACATACAGT
NGLY1	ACCGCTTCCCAAGATATAACAA	GATGGAGAATAGACTTCTGTCCAAA
NUR77	CACAGCTTGGGTGTTGATGTT	GCCCAGCAGACGTGACA
SRXN1	CAATCGCCGTGCTCAT	TGATCCAGAGGACGTCGAT
SGK1	GAATGTGAAGCACCCTTTCCT	CCCTCTGGAGATGGTAGAACA
SIAT8E	GCCCTACTACCGCTCTCAGTT	CACATCTGTGGTATATATCCCTGAGATA
SLC35A2	CCTCTCTCATCTATACCTTGCAGAATA	GTGAGAGGCTGCGATTCAA
TRP53	TGGCCATCTACAAGAAGTCACA	TCCTTCCACCCGGATAAGAT
TTR	TGACAGGATGGCTTCCCTT	AGCATCCAGGACTTTGACCAT
